# Metabotropic Glutamate Receptor 7: A New Therapeutic Target in Neurodevelopmental Disorders

**DOI:** 10.3389/fnmol.2018.00387

**Published:** 2018-10-23

**Authors:** Nicole M. Fisher, Mabel Seto, Craig W. Lindsley, Colleen M. Niswender

**Affiliations:** ^1^Department of Pharmacology and Vanderbilt Center for Neuroscience Drug Discovery, Vanderbilt University, Nashville, TN, United States; ^2^Department of Chemistry, Vanderbilt University, Nashville, TN, United States; ^3^Vanderbilt Kennedy Center, Vanderbilt University Medical Center, Nashville, TN, United States

**Keywords:** neurodevelopmental disorder, ASD, Rett syndrome, mGlu_7_, *GRM7*, allosteric modulator

## Abstract

Neurodevelopmental disorders (NDDs) are characterized by a wide range of symptoms including delayed speech, intellectual disability, motor dysfunction, social deficits, breathing problems, structural abnormalities, and epilepsy. Unfortunately, current treatment strategies are limited and innovative new approaches are sorely needed to address these complex diseases. The metabotropic glutamate receptors are a class of G protein-coupled receptors that act to modulate neurotransmission across many brain structures. They have shown great promise as drug targets for numerous neurological and psychiatric diseases. Moreover, the development of subtype-selective allosteric modulators has allowed detailed studies of each receptor subtype. Here, we focus on the metabotropic glutamate receptor 7 (mGlu_7_) as a potential therapeutic target for NDDs. mGlu_7_ is expressed widely throughout the brain in regions that correspond to the symptom domains listed above and has established roles in synaptic physiology and behavior. Single nucleotide polymorphisms and mutations in the *GRM7* gene have been associated with idiopathic autism and other NDDs in patients. In rodent models, existing literature suggests that decreased mGlu_7_ expression and/or function may lead to symptoms that overlap with those of NDDs. Furthermore, potentiation of mGlu_7_ activity has shown efficacy in a mouse model of Rett syndrome. In this review, we summarize current findings that provide rationale for the continued development of mGlu_7_ modulators as potential therapeutics.

## Introduction

Neurodevelopmental disorders are a group of conditions that present in early life and are characterized by the failure to meet typical developmental milestones. These disorders affect a significant fraction of the population: 15% of children aged 3 to 17 years old were reported to have a developmental disability in the years 2006 to 2008 (Boyle et al., 2011). The current Diagnostic and Statistical Manual of Mental Disorders (DSM-V) categorizes NDDs into six groups: intellectual disabilities (IDs), learning disorders, communication disorders, ASDs, ADHDs, and motor disorders (American Psychiatric Association, 2013). There is often overlap between these groups; for example, 31.6% of patients with ASD also fulfill the diagnostic criteria for ID (Christensen et al., 2016). In addition, NDDs are associated with many co-morbidities, including but not limited to: epilepsy, mood disorders, breathing abnormalities, sleep problems, and gastrointestinal issues (Mannion and Leader, 2013; Doshi-Velez et al., 2014). Individuals with NDDs can struggle to develop interpersonal relationships and face immense challenges in school and in the workforce. Treatment options remain limited and there is a great need to identify novel points of intervention to improve the quality of life of these patients.

A growing body of literature suggests that NDDs arise from complex interactions between the environment and the genome (van Loo and Martens, 2007; Hu et al., 2014). In some cases, NDDs can be traced to genetic abnormalities such as point mutations, gene deletions/duplications, or chromosomal rearrangements. Examples of such disorders include Down syndrome, RTT, Fragile X syndrome, and Angelman syndrome. Although a clear genetic cause is often rare, monogenetic disorders have helped to identify proteins and pathways that are required for proper neuronal development and maintenance. Interestingly, many genes that have been associated with syndromic and non-syndromic NDDs can be clustered into pathways involved in synaptic structure and function (Spooren et al., 2012; Sztainberg and Zoghbi, 2016). In this review, we focus on the metabotropic glutamate receptor 7 (mGlu_7_), a GPCR that serves as an important regulator of synaptic transmission and plasticity. We will summarize current literature suggesting the involvement of mGlu_7_ in NDDs and discuss its potential utility as a novel therapeutic target.

## Metabotropic Glutamate Receptors

mGlu_7_ is one of eight subtypes of mGlu that are expressed throughout the body. The mGlu receptors are a family of Class C GPCRs that are further divided into three groups based on their sequence homology, signaling pathways, and ligand selectivity. Group I includes mGlu_1_ and mGlu_5_, Group II includes mGlu_2_ and mGlu_3_, and Group III includes mGlu_4_, mGlu_6_, mGlu_7_, and mGlu_8_ (Niswender and Conn, 2010). Characteristic of Class C GPCRs, all mGlu receptors consist of a large *N*-terminal ligand binding domain, a cysteine-rich domain, a heptahelical domain, and a C-terminal domain; G proteins interact with intracellular loops and the C-terminus of the receptors.

The large extracellular *N*-terminal ligand binding domain consists of two lobes that sit on top of one another, similar to a Venus flytrap. This structural similarity earned it the name VFD. Glutamate, the endogenous ligand for mGlu receptors, binds to a cleft in between the two lobes of the VFD (Kunishima et al., 2000; Pin et al., 2003; Niswender and Conn, 2010). The mGlu receptors function as constitutive dimers (Pin et al., 2005; El Moustaine et al., 2012), and dimerization primarily occurs at the level of the VFDs (Kunishima et al., 2000; Jingami et al., 2003; Pin et al., 2003; Levitz et al., 2016). The VFDs can exist in three different states within the dimer: open-open, open-closed, and closed-closed. The open-open state is the inactive state, and upon glutamate binding to the cleft of the VFD, the VFD closes and receptor activation occurs. Ligand binding to one VFD results in the open-closed conformation, whereas ligand binding to both VFDs results in the closed-closed conformation (Pin et al., 2005; Muto et al., 2007). It is suggested that glutamate binding to one VFD alone is sufficient for activation (open-closed), but that full activation is achieved when both VFDs are ligand bound (closed-closed) (Kniazeff et al., 2004). Although mGlu_7_ has been historically predicted to act as a homodimer, it has also been postulated that the receptor enacts some of its function through hetero-dimerization with other receptors, such as mGlu_8_ (Doumazane et al., 2011; Kammermeier, 2015).

The cysteine-rich domain contains nine cysteine residues linked by disulfide bonds that are critical for propagating signals from the VFDs to the rest of the receptor (Rondard et al., 2006; Muto et al., 2007). After glutamate binding, signals are transduced through the cysteine-rich domain to the heptahelical domain where conformational changes allow for G protein coupling (Kunishima et al., 2000; Tateyama et al., 2004; Binet et al., 2007; Muto et al., 2007; El Moustaine et al., 2012). mGlu_7_ and the other Group III mGlu receptors couple to G_i/o_, which inhibits adenylyl cyclase activity and reduces intracellular cAMP concentrations (Niswender and Conn, 2010). Furthermore, mGlu_7_ activation can result in K^+^ influx via G_βγ_-mediated opening of GIRK ion channels, and inhibition of Ca^2+^ currents through N- and P/Q- type calcium channels (Millán et al., 2002, 2003; Martín et al., 2007).

mGlu_7_ is the most widely expressed mGlu receptor in the CNS with relatively high expression in the amygdala, hippocampus, and hypothalamus (Kinoshita et al., 1998). There are 15 splice variants of *GRM7*, six of which are predicted to be protein coding (Zerbino et al., 2018). The two major isoforms, mGlu_7a_ and mGlu_7b_, differ at their C-termini and it is hypothesized that these distinct C-terminal tails mediate different protein-protein interactions (Dev et al., 2001). While mGlu_7a_ and mGlu_7b_ are primarily expressed in the CNS (Flor et al., 1997; Corti et al., 1998; Kosinski et al., 1999), isoform specificity has been observed in peripheral tissues such as the testes, trachea, uterus, and salivary gland (Schulz et al., 2002). In the CNS, mGlu_7_ receptors are primarily localized to presynaptic active zones in neurons where they can act as auto- or hetero-receptors to inhibit the release of their endogenous ligand, glutamate, the main excitatory neurotransmitter or GABA, the main inhibitory neurotransmitter, respectively (Shigemoto et al., 1996; Cartmell and Schoepp, 2000; Dalezios et al., 2002; Somogyi et al., 2003; Niswender and Conn, 2010). Compared to the other Group III mGlu receptors, mGlu_7_ exhibits an extremely low affinity for glutamate (high μM to mM as opposed to high nM to low mM for the other Group III mGlu receptors). Because of this low affinity, it has been suggested that mGlu_7_ functions as an “emergency brake” in the case of elevated glutamate levels (Niswender and Conn, 2010). This idea is supported by the observation that mGlu_7_ knockout mice exhibit spontaneous seizures under certain contexts (Sansig et al., 2001).

## Current mGlu_7_ Tool Compounds

**FIGURE 1:**
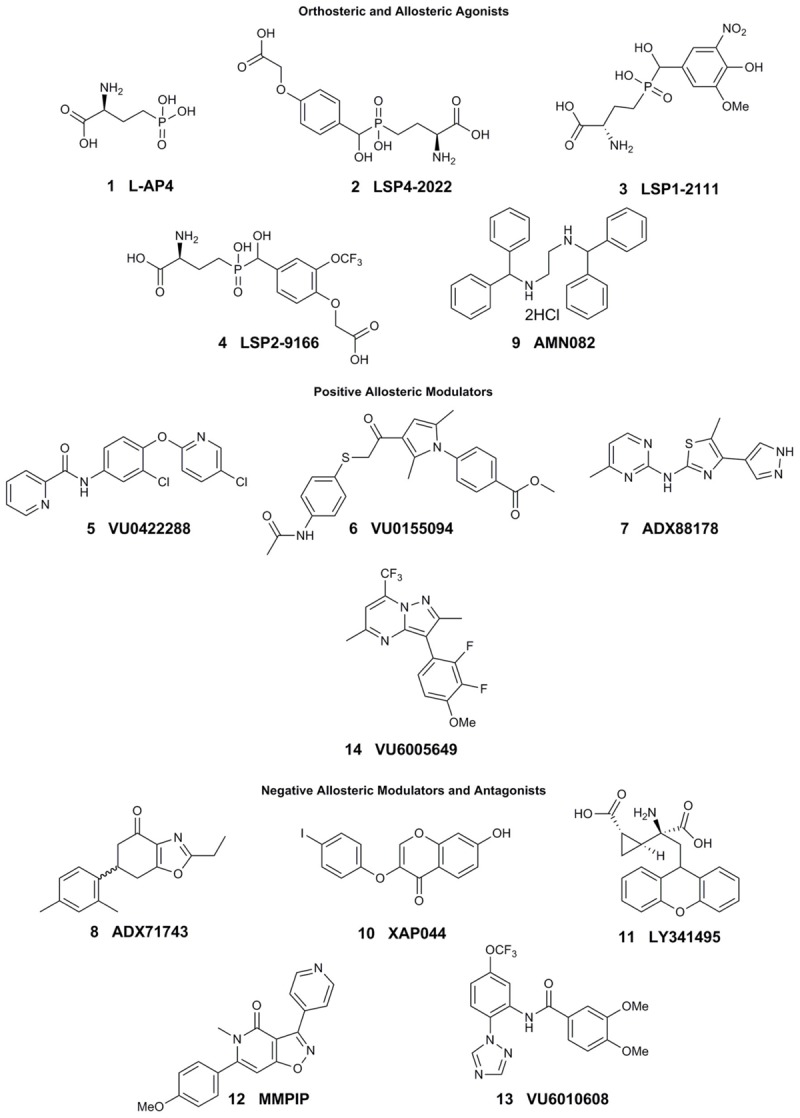
Current tool compounds used to study mGlu_7_.

Research to investigate mGlu_7_ biology has been limited, in part, due to the lack of selective tool compounds. Many of the currently existing compounds do not demonstrate high selectivity, desired pharmacokinetic properties, and/or high potency. Here, we review compounds currently available that will be mentioned in subsequent sections (compound properties at Group III mGlu receptors listed in Table 1 and structures in Figure 1).

**Table 1:** Summary of current tool compounds used to study mGlu_7_.

Name (#)	Type	mGlu_7_ pEC_50_/pIC_50_	mGlu_8_ pEC_50_/pIC_50_	mGlu_4_ pEC_50_/pIC_50_	mGlu_6_ pEC_50_/pIC_50_	Source
L-AP4 **(1)**	Orthosteric agonist	3.47 (PIH)	6.53 (PIH)	7.00 (PIH)	5.62 (PIH)	Acher et al., 2012; Selvam et al., 2018
		3.61 (Ca^2+^)	6.53 (Ca^2+^)	6.89 (Ca^2+^)	6.00 (Ca^2+^)	
LSP4-2022 **(2)**	Orthosteric agonist	4.34 (Ca^2+^)	4.54 (Ca^2+^)	6.96 (Ca^2+^)	5.36 (Ca^2+^)	Acher et al., 2012; Goudet et al., 2012; Selvam et al., 2018
LSP1-2111 **(3)**	Orthosteric agonist	4.28 (PIH)	4.18 (PIH)	5.66 (PIH)	5.77 (PIH)	Selvam et al., 2018
		4.00 (Ca^2+^)	4.71 (Ca^2+^)	6.05 (Ca^2+^)	5.49 (Ca^2+^)	
LSP2-9166 **(4)**		5.71 (Ca^2+^)	4.25 (Ca^2+^)	7.22 (Ca^2+^)	Not reported	Acher et al., 2012
VU0422288 **(5)**	Group III PAM	6.85 (Ca^2+^)	6.93 (Ca^2+^)	6.98 (Ca^2+^)	Not reported	Jalan-Sakrikar et al., 2014
VU0155094 **(6)**	Group III PAM	5.80 (Ca^2+^)	6.07 (Ca^2+^)	5.48 (Ca^2+^)	Not reported	Jalan-Sakrikar et al., 2014
ADX88178 **(7)**	mGlu_4/8_ PAM	>4.52 (Ca^2+^)	5.66 (Ca^2+^)	8.46 (Ca^2+^)	>5	Le Poul et al., 2012
ADX71743 **(8)**	mGlu_7_ NAM	7.20 (human, Ca^2+^)	Inactive	Inactive	Inactive	Kalinichev et al., 2013
		7.06 (rat, Ca^2+^)	Inactive	Inactive	Inactive	
AMN082 **(9)**	Allosteric agonist	6.59 (GTPγS)	>5 (GTPγS)	>5 (GTPγS)	>5 (GTPγS)	Mitsukawa et al., 2005
XAP044 **(10)**	Antagonist	5.26 (cAMP)	4.48 (cAMP)	Inactive	Inactive	Gee et al., 2014
		5.55 to 5.46 (GTPγS)				
LY341495 **(11)**	Orthosteric antagonist	6.00 (cAMP)	6.76 (cAMP)	4.66 (cAMP)	Not reported	Kingston et al., 1998
MMPIP **(12)**	mGlu_7_ NAM	6.66 (cAMP)	>5 (cAMP)	>5 (cAMP)	Not reported	Suzuki et al., 2007
		7.15 (Ca^2+^)				Niswender et al., 2010
		6.14 (Thallium)				Niswender et al., 2010
VU6010608 **(13)**	mGlu_7_ NAM	6.12 (Ca^2+^)	>5 (Ca^2+^)	>5 (Ca^2+^)	Inactive (>5)	Reed et al., 2017
VU6005649 **(14)**	mGlu_7/8_ PAM	6.19 (Ca^2+^)	5.59 (Ca^2+^)	>5 (Ca^2+^)	Inactive	Abe et al., 2017


*NAM, negative allosteric modulator; PAM, positive allosteric modulator; EC_*50*_, effective concentration 50; IC_*50*_, inhibitory concentration 50. Assay type is indicated in parenthesis: PIH, phosphatidylinositol hydrolysis; cAMP, cAMP accumulation; Ca^*2*+^, calcium mobilization; GTPγS, GTPγS binding.*

The development of mGlu_7_ PAMs and other activators has been a major challenge thus far. Many *in vitro* and *in vivo* studies examining the effects of mGlu_7_ potentiation have been performed with orthosteric Group III mGlu agonists such as L-2-amino-4-phosphonobutyric acid (L-AP4, **1**), LSP4-2022 (**2**), and LSP1-2111 (**3**). L-AP4 exhibits an *in vitro* potency (EC_50_) of 0.1, 337, and 0.29 μM at mGlu_4_, mGlu_7_, and mGlu_8_, respectively (Acher et al., 2012; Selvam et al., 2018). Similarly, LSP4-2022 exhibits *in vitro* EC_50s_ of 0.11, 11.6, and 29.2 μM at mGlu_4_, mGlu_7_, and mGlu_8_, respectively (Acher et al., 2012; Goudet et al., 2012; Selvam et al., 2018), while a structurally-related analog, LSP1-2111, displays EC_50s_ of 2.2, 53, and 66 μM at each of these receptors (Selvam et al., 2018). In addition to their relatively low potency at mGlu_7_, these orthosteric agonists have activity at the other Group III mGlu receptors, limiting their utility for the specific exploration of mGlu_7_ biology. Interestingly, the orthosteric mGlu_4/7_-preferring agonist LSP2-9166 is much more potent at mGlu_7_ compared to the other agonists described above (EC_50s_ = 0.06, 1.97, 55.6 μM at mGlu_4_, mGlu_7_, and mGlu_8_), but has yet to be investigated further in learning and memory paradigms (Acher et al., 2012; Hajasova et al., 2018; Lebourgeois et al., 2018).

Pan-Group III PAMs such as VU0422288 (**5**), which exhibits EC_50s_ of 108, 146, and 125 nM, for mGlu_4_, mGlu_7_, and mGlu_8_, respectively, and VU0155094 (**6**), 3.2, 1.5, and 0.9 μM (Jalan-Sakrikar et al., 2014) are also used. Additionally, VU6005649 **14**) is a dual mGlu_7/8_ PAM, with EC_50_ values of 650 nM and 2.6 μM at mGlu_7_ and mGlu_8_, respectively. In addition to its activity on mGlu_8_, VU6005649 displays off-target effects at the neurokinin-1 receptor (NK1). It is believed that these effects on NK1 may mediate sedative effects of this compound, which are observed in both wild-type and mGlu_7_ knockout animals (Abe et al., 2017). Because many of these tool compounds are not selective, they have been used concomitantly with other molecules, such as the mGlu_4/8_ PAM ADX88178 (**7**) or mGlu_7_ NAM ADX71743 (**8**), to confirm mGlu_7_-mediated effects (Le Poul et al., 2012; Kalinichev et al., 2013, 2014; Gogliotti et al., 2017).

To date, only one mGlu_7_-selective allosteric agonist, AMN082 (**9**, EC_50_ = 260 nM), has been reported in the primary literature (Mitsukawa et al., 2005). AMN082 has been used for animal studies involving learning and memory and plasticity in the amygdala among other areas. However, it has been shown that AMN082 exhibits off-target effects, one of which is predicted to be inhibition of the serotonin transporter (SERT) (Sukoff Rizzo et al., 2011; Ahnaou et al., 2016), somewhat limiting its utility *in vivo* unless coupled with knockout studies.

In contrast to potentiators, there have been several mGlu_7_ selective antagonists and NAMs reported in the literature. The antagonist XAP044 (**10,** IC_50_ = 5.5 μM) binds within the VFD and has shown efficacy in both *in vivo* and *in vitro* experiments such as anxiety-, depression-, and fear-related behavioral tasks and electrophysiology (Gee et al., 2014). Originally labeled a Group II mGlu receptor antagonist (mGlu_2_ and mGlu_3_), LY341495 (**11**) was also found to have efficacy at mGlu_4_, mGlu_7_, and mGlu_8_ with IC_50s_ of 22, 0.99, and 0.173 μM, respectively (Kingston et al., 1998) and has been used to study both groups of mGlu receptor since its discovery.

The mGlu_7_ NAM 6-(4-methoxyphenyl)-5-methyl-3-pyridin-4-ylisoxazolo[4,5-*c*]pyridin-4(5*H*)-one (MMPIP, **12**), reported in 2007, has been used for several studies involving mGlu_7_ (Suzuki et al., 2007). MMPIP is able to inhibit the response of L-AP4, but its efficacy was later shown to be context-dependent. For example, the potency (IC_50_) of MMPIP was 70 nM in a calcium mobilization assay utilizing cells expressing G_α15_ versus 718 nM in a thallium flux assay with cells expressing G_αi/o_, suggesting that its effects may be dependent on cellular background. Further, MMPIP was not effective in blocking an mGlu_7_-mediated depression of synaptic transmission in electrophysiological studies (Niswender et al., 2010). ADX71743, reported in 2013, exhibits an IC_50_ of 63 and 88 nM at human and rat mGlu_7_, respectively (Kalinichev et al., 2013). However, it also exhibits low activity at mGlu_2_ (Kalinichev et al., 2013; Reed et al., 2017) and possesses an electrophilic ketone moiety that could result in covalent modification and subsequent off-target effects. Most recently, Reed et al. (2017) have successfully developed a series of novel, chemically-distinct mGlu_7_ NAMs based upon a phenylbenzamide scaffold. One of the analogs, VU6010608 (**13**), exhibited modest potency (IC_50_ = 759 nM), but was cleared rapidly in rats (64.2 mL/min/kg) and exhibited low levels of brain penetration, making it challenging for *in vivo* CNS studies (Reed et al., 2017). These existing and emerging tools, coupled with mGlu_7_ knockout mice, have provided an initial toolbox to begin elucidation of the function of mGlu_7_ in normal and pathological conditions.

## mGlu_7_ in Synaptic Transmission and Plasticity

Inhibition of neurotransmitter release by mGlu_7_ is believed to be mediated by the inhibition of N-type and P/Q-type calcium channels through interactions with G_βγ_, PKC, PICK1, and reductions in intracellular cAMP (Millán et al., 2002, 2003; Perroy et al., 2002; Martín et al., 2007). Millan and Colleagues demonstrated that activation of cerebrocortical mGlu_7_ with L-AP4 inhibited N-type calcium channels in a PKA- and PKC-independent manner, suggesting that the inhibition was caused via interactions with G_βγ_. These authors also demonstrated that mGlu_7_-mediated decreases in cAMP could reduce spontaneous glutamate release in the cerebral cortex (Millán et al., 2002). Additionally, Perroy et al. (2000) demonstrated that P/Q-type calcium channels were inhibited via a PKC-dependent pathway, where G_i/o_ and/or G_βγ_ can stimulate the PLC pathway in cultured cerebellar granule cells. They also showed that the scaffolding protein, PICK1, facilitates the interaction between mGlu_7_ and PKC, and is required for receptor-mediated P/Q-type calcium channel inhibition in this context (Perroy et al., 2002). In contrast, Martín et al. (2007) demonstrated that mGlu_7_ inhibited hippocampal P/Q-type calcium channels in a PKC-independent manner. The mGlu_7_-mediated inhibition of glutamate release is also dependent on interactions with calmodulin (CaM), where activated CaM allows for the displacement of G_βγ_ from mGlu_7_ and the subsequent downregulation of calcium influx into the cell via calcium channel inhibition (O’Connor et al., 1999). Moreover, mGlu_7_’s interaction with MacMARCKS (macrophage myristoylated alanine–rich C–kinase substrate) competitively antagonizes CaM-mediated calcium channel inhibition (Bertaso et al., 2006).

mGlu_7_’s position within the active zone and its ability to modulate neurotransmitter release has led to numerous studies focused on its role in synaptic plasticity. Two major forms of synaptic plasticity include LTP and LTD, which are persistent changes in synaptic strength that are thought to be correlates of learning and memory (Bliss and Collingridge, 1993; Takeuchi et al., 2014). The role of mGlu_7_ in synaptic plasticity has been best characterized within the hippocampus at several distinct synapses. mGlu_7_ was first reported to mediate a form of LTD occurring in stratum radiatum interneurons within area CA3 (Laezza et al., 1999). At excitatory synapses onto interneurons expressing calcium-permeable AMPA receptors, LTP could be induced by high frequency stimulation and blocked by the Group II and Group III mGlu antagonist, LY341495. Further pharmacological experiments confirmed the specific involvement of mGlu_7_: only a high concentration of L-AP4 depressed synaptic transmission at these synapses and a Group II mGlu agonist showed no effect. A similar form of plasticity was later described at mossy fiber inputs onto SLINs in area CA3 (Pelkey et al., 2005). At SLINs expressing calcium-permeable AMPA receptors, high frequency stimulation of mossy fibers induced an LTD that required mGlu_7_ activation and PKC-dependent depression of neurotransmitter release through P/Q-type voltage gated calcium channels (Pelkey et al., 2005, 2006). Interestingly, in slices pre-treated with L-AP4, internalization of mGlu_7_ receptors revealed the ability of these synapses to undergo LTP instead of LTD in response to the same electrical stimulus. Surface expression of mGlu_7_, therefore, regulates the direction of plasticity at these synapses, making mGlu_7_ a “metaplastic switch” that can modulate feedforward inhibition in area CA3.

An additional class of interneurons in which mGlu_7_-mediated plasticity has been implicated is the OLM interneuron population within the stratum oriens of areas CA3 and CA1. At excitatory inputs onto OLM interneurons, mGlu_7_ expression is preferentially enriched (Shigemoto et al., 1996) and proposed to be recruited by extracellular-leucine-rich repeat fibronectin type III domain containing 1, or ELFN1 (Tomioka et al., 2014). Sylwestrak and Ghosh demonstrated that ELFN1 knockdown in OLM interneurons decreases short-term facilitation and increases presynaptic release probability. Conversely, overexpression of ELFN1 in parvalbumin interneurons leads to short-term facilitation when these synapses typically undergo short-term depression (Sylwestrak and Ghosh, 2012). In slices from *Elfn1^-/-^* mice, presynaptic release probability, short term facilitation, and LTP are reduced in patch-clamp recordings from OLM interneurons (Tomioka et al., 2014). Although this evidence is indirect, it suggests that mGlu_7_ may be involved in these forms of synaptic plasticity since mGlu_7_ is likely to be a major regulator of presynaptic release probability at these synapses due to its recruitment by ELFN1.

In addition to its role as an autoreceptor on excitatory terminals, mGlu_7_ is also located on the terminals of interneurons within the hippocampus and modulates the release of GABA (Somogyi et al., 2003; Summa et al., 2013). This function of mGlu_7_ is required for LTP in wild-type animals at SC-CA1 synapses through a mechanism of disinhibition (Klar et al., 2015). Importantly, deficits in LTP at this particular synapse have been reported in several models of NDDs (Jiang et al., 1998; Moretti et al., 2006; von der Brelie et al., 2006). At SC-CA1 synapses, mGlu_7_ is the only presynaptic mGlu receptor present in adult animals and activation of mGlu_7_ has been repeatedly shown to reduce field potentials at SC-CA1 (Baskys and Malenka, 1991; Ayala et al., 2008; Jalan-Sakrikar et al., 2014). Klar et al. (2015) demonstrated that mGlu_7_ activation by the agonist LSP4-2022 also reduces evoked inhibitory post-synaptic currents recorded from CA1 pyramidal cells. LTP induced by high-frequency stimulation was blocked by ADX71743, but only when GABAergic transmission was intact. Recently, we showed that a chemically distinct mGlu_7_ NAM, VU6010608, also blocked LTP induced by high-frequency stimulation at SC-CA1 synapses (Reed et al., 2017). Interestingly, hippocampal slices from *Grm7^-/-^* mice have been reported to exhibit similar levels of LTP when compared to WT controls, but decreased short-term potentiation following high-frequency stimulation (Bushell et al., 2002). In these studies, slices from *Grm7^-/-^* mice showed reduced facilitation during the high-frequency train, an effect that was also seen with ADX71743 by Klar et al. (2015). The presence of LTP in *Grm7^-/-^* slices may be due to compensatory mechanisms during development, such as retained expression of mGlu_8_, which is present at SC-CA1 synapses earlier in development (Ayala et al., 2008). Re-expression of mGlu_8_ is not unprecedented as the selective mGlu_8_ agonist (S)-3,4-DCPG was recently shown to reduce synaptic transmission at SC-CA1 in slices from pilocarpine-treated rats, but not in those of age-matched controls (Dammann et al., 2018). While further studies will be needed to explain the current discrepancy between genetic and pharmacological approaches, these data indicate that mGlu_7_ regulates high-frequency transmission at SC-CA1 synapses. Recently, Martín et al. (2018) demonstrated that prolonged activation of mGlu_7_ leads to potentiation of excitatory post-synaptic currents recorded by pyramidal cells in CA1. This potentiation of neurotransmitter release is dependent on PLC and the vesicle release proteins Munc13-2 and Rim1α. These studies indicate that, under conditions of high-frequency stimulation, mGlu_7_ activation favors potentiation of excitatory transmission, which could be an additional mechanism by which mGlu_7_ modulates long-term plasticity in the hippocampus.

Beyond the hippocampus, a role for mGlu_7_ in LTP has also been established within the amygdala. Synaptic plasticity in the hippocampus is believed to underlie associative learning and working memory, whereas plasticity in the amygdala is associated with aversion and emotional learning (Brasted et al., 2003; Sigurdsson et al., 2007). The allosteric agonist AMN082 has been shown to block LTP at thalamo-amygdala synapses in slices from rats and mice (Fendt et al., 2008, 2013). This effect correlates with the ability of direct injection of AMN082 into the amygdala to block the acquisition of fear-potentiated startle behavior in rats (Fendt et al., 2008) and fear learning in mice (Fendt et al., 2008, 2013). Interestingly, *Grm7^-/-^* mice exhibit a general deficit in fear learning and decreased LTP at thalamo-amygdala synapses (Fendt et al., 2013). Reduction of LTP by both an agonist and gene ablation may be explained by AMN082’s ability to cause rapid internalization of mGlu_7_ receptors (Pelkey et al., 2007). This would suggest that AMN082 can act as a functional antagonist by decreasing surface expression and, therefore, receptor signaling. This hypothesis is further supported by the ability of the mGlu_7_ antagonist XAP044 to block LTP within the amygdala, inhibit acquisition of conditioned fear, and reduce anxiety-like behavior (Gee et al., 2014). Together, these studies demonstrate that mGlu_7_ promotes plasticity within the amygdala, which is in line with its involvement in behaviors of fear and anxiety.

## Role of mGlu_7_ in NDD-Associated Phenotypes

Core symptoms and comorbidities of NDDs can include, but are not limited to: cognitive impairment, seizures, mood disorders, social deficits, and motor impairments (Mannion and Leader, 2013; Doshi-Velez et al., 2014). Many studies have demonstrated that modulation of mGlu_7_ function via genetic and/or pharmacologic techniques is able to mimic some of these phenotypes in animal models, and these studies will be reviewed here.

## Cognition

mGlu_7_ knockout animals (*Grm7^-/-^*) show deficits in tasks that test cognitive functioning. In a conditioned taste aversion task, which measures amygdala-dependent aversive learning, mice were given saccharin along with an intraperitoneal injection of the control, saline, or LiCl, which evokes malaise. In this task, *Grm7^-/-^* mice did not associate the adverse effects of LiCl to saccharin in comparison to wild-type littermates, exhibiting a deficit in fear learning (Masugi et al., 1999). In addition, Masugi et al. (1999) and Goddyn et al. (2008, 2015) demonstrated that *Grm7^-/-^* mice exhibit less freezing than wild-type animals in cued and contextual fear conditioning paradigms. Together, these results indicate a role for mGlu_7_ in aversion learning, and also suggest that the loss of mGlu_7_ causes impairments in these learning paradigms.

mGlu_7_ has also been demonstrated to play a role in cognitive tasks that do not rely on fearful or aversive stimuli. Callaerts-Vegh et al. (2006) showed that *Grm7^-/-^* mice exhibit impaired short-term working memory in 4- and 8-arm radial maze tasks, committing more errors (visits to previously baited arms or un-baited arms) than their wild-type counterparts. Conversely, *Grm7^-/-^* mice performed similarly to wild-type animals in radial maze tasks when they were modified to assess long-term memory. Furthermore, both Callaerts-Vegh et al. (2006) and Goddyn et al. (2015) have reported that the loss of mGlu_7_ causes increased latency to locate a platform in the Morris water maze task of spatial memory. Interestingly, *Grm7^-/-^* mice performed similarly to wild-type animals after increased training and in un-cued trials (Callaerts-Vegh et al., 2006). Together, these data demonstrate that mGlu_7_ may play specific roles in tasks involving working and spatial memory.

Pharmacological studies have further confirmed a role for mGlu_7_ in learning and memory. Hikichi et al. (2010) showed that administration of MMPIP, an mGlu_7_ NAM, to wild-type mice reduced performance in object recognition and location tasks, suggesting that mGlu_7_ is also involved in recognition memory. MMPIP also attenuates conditioned taste aversion learning in rats (Klakotskaia et al., 2013). Interestingly, MMPIP improved cognitive performance in Y-maze and object recognition assays in a mouse model of neuropathic pain with no effect on sham-treated animals (Palazzo et al., 2015). As discussed above, MMPIP exhibits cellular background-dependent differences *in vitro*, and also had no effect in an electrophysiological study of at SC-CA1 synapses in the hippocampus (Niswender et al., 2010), which may complicate interpretation of *in vivo* data. Inhibition of mGlu_7_ with the antagonist XAP044 also resulted in reduced freezing in mice during a contextual fear conditioning task, further supporting a role for mGlu7 in amygdala function (Gee et al., 2014). Activation of mGlu_7_ with an allosteric agonist, AMN082, has been shown to modulate both the acquisition and extinction of conditioned fear, though the results seem to contradict findings from studies performed with XAP044 and *Grm7^-/-^* animals (Fendt et al., 2008, 2013; Goddyn et al., 2008; Siegl et al., 2008; Dobi et al., 2013; Gee et al., 2014). Administration of AMN082 impairs the acquisition and enhances the extinction of fear learning (Fendt et al., 2008, 2013; Siegl et al., 2008; Dobi et al., 2013), but knockout animals exhibit similar phenotypes in conditioned fear paradigms (Goddyn et al., 2008; Fendt et al., 2013). AMN082 appears to exhibit a task-dependent phenotype, where mGlu_7_ activation facilitates between-session extinction, but not within-session extinction in a fear conditioning model (Toth et al., 2012; Fendt et al., 2013). AMN082 was also shown to have effects in social fear; it impaired extinction and recall when administered prior to the social fear extinction task, but not when given before social fear conditioning (Slattery et al., 2017). However, Ahnaou et al. (2016) demonstrated that AMN082 produced similar sleep-wake and hypothermia phenotypes in *Grm7^-/-^* and wild-type mice, suggesting that there may be off-target effects elicited by the compound. Additionally, administration of VU6005649, an mGlu_7/8_ PAM, to wild-type mice, increases freezing in contextual fear conditioning (Abe et al., 2017).

## Seizures

Seizures are often present in patients with NDDs, and mGlu_7_ and its interacting proteins have been implicated in seizure activity. Sansig et al. (2001) observed that *Grm7^-/-^* mice suffered from spontaneous sensory stimulus-seizures and were also more susceptible to subconvulsant doses of PTZ and bicuculline than their heterozygous or wild-type littermates. In addition, reduction of mGlu_7_ activity with the NAM ADX71743 was sufficient to induce absence seizures (Tassin et al., 2016). Disruption of proteins that interact with mGlu_7_ can also induce seizures in mice (Bertaso et al., 2008; Tomioka et al., 2014). For example, PICK1 is a PDZ-domain containing protein that interacts with the C-terminus of mGlu_7_. The protein-protein interaction between PICK1 and mGlu_7_ is important for stable mGlu_7_ cell surface expression, proper trafficking of mGlu_7_ to presynaptic active zones, and also for inhibition of P/Q-type calcium channels. Disruption of the interaction between PICK1 and mGlu_7_ appears to interfere with mGlu_7_’s inhibitory activity via decreased cell surface stability/expression or improper signaling and trafficking, resulting in a seizure phenotype in mice (Perroy et al., 2002; Bertaso et al., 2008; Zhang et al., 2008).

As mentioned previously, ELFN1 is a transmembrane protein that has been demonstrated to recruit mGlu_7_ to distinct cell populations in the hippocampus and cortex (Tomioka et al., 2014). Most recently, ELFN1 was also shown to be a trans-synaptic allosteric modulator of Group III mGlu receptors; receptor modulation occurs through an ELFN1-mediated alteration of G-protein coupling efficiency to the Group III mGlu receptors (Dunn et al., 2018). Of note, ELFN1 mutations clustered in the region required for mGlu_7_ recruitment have been found in patients with epilepsy and ADHD (Dolan and Mitchell, 2013; Tomioka et al., 2014), and ELFN1 knockout (*Elfn1*^-/-^) animals exhibit a similar seizure phenotype to *Grm7*^-/-^ animals (Tomioka et al., 2014). Interestingly, *Elfn1*^-/-^ mice also exhibit ADHD-like phenotypes such as hyperactivity and impulsivity. Dolan and Mitchell (2013) showed that *Elfn1*^-/-^ animals display hyperlocomotion and increased activity in an open field. Administration of amphetamine to *Elfn1*^-/-^ mice was able to attenuate hyperlocomotion, similar to the effects of stimulant therapies for ADHD patients. Tomioka et al. (2014) also demonstrated that *Elfn1*^-/-^ mice displayed more spontaneous activity than wild-type animals and also exhibited decreased immobility in a forced swim test, which are behaviors suggestive of hyperactivity. *Elfn1*^-/-^ mice spent more time in the open arms during an EPM task compared to wild-type littermates. These data are typically indicative of anxiolytic effects; however, *Elfn1*^-/-^ mice showed no preferences between the light and dark boxes of the light-dark box transition task. Based on this finding, the authors hypothesized that the results of the EPM were indicative of impulsivity. Together, these data suggest a role for the ELFN1-mGlu_7_ complex in seizures and in other disorders.

## Mood Disorders

mGlu_7_ modulation has also been demonstrated to impact behavioral models of mood disorders such as anxiety or depression, which are common comorbidities seen in NDDs (Matson and Cervantes, 2014). The amygdala and hippocampus, areas of high mGlu_7_ expression, are brain regions known for their importance in anti-anxiety and anti-depressive action (Shin and Liberzon, 2010). In comparison to cognitive tasks, where reductions in mGlu_7_ cause deficits, the loss of mGlu_7_ has been reported to result in anti-depressive and anxiolytic effects in these domains. For example, Cryan et al. (2003) showed that *Grm7*^-/-^ animals spend more time in the open arms than their wild-type counterparts in an EPM paradigm, demonstrating that the loss of the receptor causes anxiolytic activity. In a light-dark box task, the knockout animals have a reduced latency to enter a covered, dark compartment as well as an increased number of transitions into an open, brightly lit compartment than wild-type mice (Cryan et al., 2003). Callaerts-Vegh et al. (2006) demonstrated that *Grm7*^-/-^ mice bury fewer marbles than wild-type animals in a marble burying task, which also measures anxiety-like behavior in rodents. ADX71743, the mGlu_7_-selective NAM, causes similar results in EPM, and reduces marble burying in wild-type mice (Kalinichev et al., 2013). Administration of the NAM MMPIP also reduces marble burying, consistent with the *Grm7*^-/-^ phenotype (Palazzo et al., 2015). In tail suspension or forced swim tasks, where immobility is indicative of depression-like behavior, *Grm7*^-/-^ mice are less immobile than wild-type animals (Cryan et al., 2003). In wild-type mice, the antagonist XAP044 also increases time in open arms in EPM and decreases immobility in tail suspension, recapitulating data from studies using knockout animals (Gee et al., 2014). In a mouse model of neuropathic pain, the NAM MMPIP also reduces immobility time during tail suspension (Palazzo et al., 2015). The mGlu_7_ agonist AMN082 reduces immobility in tail suspension and forced swim tasks, and MMPIP can block the effect of AMN082 (O’Connor and Cryan, 2013; Pałucha-Poniewiera and Pilc, 2013). In summary, mGlu_7_ has been implicated in a range of behaviors in rodent models, many of which mimic those reported in rodent models of NDDs.

## Genetic Associations Between mGlu_7_ and NDDs

Genetic associations between NDDs and *GRM7*, the gene that encodes mGlu_7_ in humans, provide a link between experiments in rodent models and the clinical population. ASD affects as much as 1% of the world’s population (Lai et al., 2014), and family studies have suggested that the heritability of ASD is about 83% (Sandin et al., 2017), which indicates a strong genetic component. Heterozygous deletions in *GRM7* have been identified in three ASD patients by Gai et al. (2012), and in one patient by Liu et al. (2015). The latter patient exhibited language and cognitive impairments as well as hyperactivity, stereotyped behaviors, and deficits in social interaction (Liu et al., 2015). An additional ASD patient with a *de novo* point mutation in *GRM7*, resulting in a change from arginine to glutamate at amino acid 622, was reported by Sanders et al. (2012). This mutation affects the third transmembrane portion of the receptor. Yang and Pan (2013) identified the SNPs rs6782011 and rs779867, which encode a C to T change in intron 6 and a T to C or T to G change in intron 5 in *GRM7*, respectively. These two polymorphisms exhibited significant associations with ASD from a group of 22 ASD patients (Yang and Pan, 2013). In an Iranian cohort of 518 ASD patients, however, only rs779867 was identified as a SNP that associates *GRM7* with ASD (Noroozi et al., 2016). rs779867 is a T to C or T to G polymorphism in intron 5 hypothesized to have effects on a MRG protein binding motif. MRG motif-binding proteins are thought to bind chromatin and function in the regulation of gene transcription (Chen et al., 2010).

Attention deficit hyperactivity disorder is characterized by inattention, hyperactivity and impulsivity (American Psychiatric Association, 2013). Its estimated prevalence around the world is 7.2% in children and 3.4% in adults (Fayyad et al., 2007; Thomas et al., 2015). A genome wide copy-number variation study revealed that rs7623055, which encodes a G to C or G to T change, was significantly associated with ADHD, and also identified six different deletions in *GRM7* in patients with ADHD (Elia et al., 2011). Additionally, rs37952452 was found to have some association with ADHD in a study of 202 patients in Korea, though it was not significantly associated when using a case-control approach (Park et al., 2013). In contrast, neither rs37952452 nor rs7623055 were found to be significantly associated with ADHD in a later study (Akutagava-Martins et al., 2014). Interestingly, ADHD patients with the G/A genotype of rs37952452 showed an improved response to methylphenidate in comparison to those with the G/G genotype (Park et al., 2014).

Rare mutations in *GRM7* have also been implicated in undiagnosed NDDs. Whole-exome sequencing in 31 consanguineous Arab families with developmental delay and/or intellectual disability revealed two families with mutations in *GRM7*. Two brothers in the same family were homozygous for a 461T/C variant, which results in the missense mutation I154T in the ligand binding domain of mGlu_7_. The same study also identified two siblings (brother and sister) who are compound heterozygous for the mutations 1972C/T and 2024C/A, which lead to missense mutations, R658W and T675Y, respectively, in the third transmembrane domain. These four patients share symptoms that include developmental delay, ID, brain malformations and seizures (Charng et al., 2016). In a different set of consanguineous families, exome sequencing identified two female cousins with the homozygous mutation 1757G/A, which results in a premature truncation of mGlu_7_ prior to its first transmembrane domain (W568^∗^). These patients exhibit seizures, profound ID, microcephaly and leukodystrophy (Reuter et al., 2017). A search of the DECIPHER database (Firth et al., 2009) identified 69 patients with a deletion or duplication that included *GRM7*, although most of these also affected other genes. Three of these patients had a deletion or duplication restricted to the *GRM7* gene and their phenotypes are included in Table 2.

**Table 2:** Summary of *GRM7* mutations identified in NDD patients.

Type	Chromosome 3 position	Nucleotide/protein change NM_00844.3	Location in transcript NM_00844.3	Zygosity	Phenotype	Source
Duplication	6209671–6981117		5′ UTR and Exon 1	Heterozygous	Behavioral abnormality, ID	DECIPHER 289768
Point mutation	6861849	c.T461T > C p.I154T	Exon 1	Homozygous	Developmental delay, seizures, hypotonia, atrophy, thin corpus callosum	Charng et al., 2016
Deletion	7053179–7144453		Intron 1/2 and Exon 2	Heterozygous	ASD	Gai et al., 2012
Deletion	70664629–7172715		Exon 2	Heterozygous	ASD	Gai et al., 2012
Deletion	7065422–7172715		Exon 2	Heterozygous	ASD	Gai et al., 2012
Deletion	7257514–7442882		Exons 3–5	Heterozygous	Global developmental delay	DECIPHER 356330
Deletion	7221090–7524552		Exons 3–7	Heterozygous	ASD	Liu et al., 2015
Point mutation	7578663	c.1757 G > A p.W586^∗^	Exon 8	Homozygous	Developmental delay, ID, microcephaly, seizures, leukodystrophy	Reuter et al., 2017
Point mutation	7578771	c.1865 G > A p.R622Q	Exon 8	Heterozygous	ASD	Sanders et al., 2012
Point mutation	7578878, 7578930	c.1972C > T p.R658W, c.2024C > A p.T675K	Exon 8	Compound Heterozygous	Developmental delay, ID, hypotonia, hypomyelination, brain atrophy, seizures	Charng et al., 2016
Duplication	7509664–7878406		Exons 8–10	Heterozygous	ID, microcephaly	DECIPHER 288108


## mGlu_7_ in *MECP2*-Related Disorders

Preclinical research in the NDD field has focused largely on mouse models of genetic syndromes due to their high construct validity. RTT is a monogenetic disorder in which mGlu_7_ has recently gained particular interest as a potential therapeutic target (Gogliotti et al., 2017). RTT is a debilitating NDD affecting 1 in 20,000 births and is characterized by a period of normal development followed by sudden developmental regression and loss of acquired skills at 6 to 18 months of age. Following regression, RTT patients are burdened by life-long symptoms that include repetitive hand clasping, limited speech, intellectual disability, motor impairment, apneas, and epilepsy (Neul et al., 2010). The majority of RTT cases can be attributed to loss-of-function mutations in the X-linked gene *MECP2*, which encodes the transcriptional regulator methyl-CpG binding protein 2 (MeCP2) (Amir et al., 1999). Since this discovery, nearly two decades of research have yielded significant insight into the functions of MeCP2 within the brain. Of note, *MECP2* mutations have also been identified in patients with ASD and ID independent of a RTT diagnosis (Couvert et al., 2001; Carney et al., 2003), suggesting that pathways involving MeCP2 may underlie NDDs more broadly. MeCP2 is canonically thought to repress gene transcription through binding to methylated CpG dinucleotides and recruiting repressor complexes; however, MeCP2 has also been shown to activate gene transcription and play roles in long-range regulation of chromatin structure, mRNA splicing and micro-RNA processing (Guy et al., 2011). Although MeCP2 is involved in prenatal and postnatal development (Tate et al., 1996; Shahbazian et al., 2002; Bedogni et al., 2016), phenotypes of *Mecp2* knockout mice can be reversed if *Mecp2* expression is reintroduced in adult animals (Guy et al., 2007). Similarly, ablation of *Mecp2* expression in adult mice following normal development is sufficient to recapitulate the phenotype of constitutive *Mecp2* knockout mice (McGraw et al., 2011). MeCP2 is thus critical for proper neuronal function throughout life and there exists a therapeutic window to improve disease severity, even at adult stages. These proof-of-concept studies have fueled programs to develop *MECP2* replacement strategies, along with parallel efforts to identify targets downstream of MeCP2 dysfunction that may be amenable to pharmacological manipulation.

mGlu_7_ is one of three mGlu receptors found to be decreased at the mRNA level in a RTT mouse model (Bedogni et al., 2016). These mGlu receptors represent a potential point of access to normalize synaptic function in RTT. Consistent with this initial report, we have shown that mGlu_7_ protein expression is significantly decreased in motor cortex autopsy samples from RTT patients compared to those of controls matched for age, sex, and postmortem interval (Gogliotti et al., 2017). In global *Mecp*2 knockout mice, mGlu_7_ protein expression is decreased in a brain-region specific manner with a notable reduction in hippocampal synaptosomal fractions. This correlates with reduced depression of synaptic transmission at SC-CA1 synapses by LSP4-2022 in slices from RTT model mice, which can be restored by a PAM. Additionally, pre-application of two structurally distinct Group III mGlu receptor PAMs, VU0422288 and VU0155094, to slices was able to restore deficient LTP at SC-CA1 synapses in RTT model mice. Ablation of *Mecp2* selectively from GABAergic neurons is sufficient for LTP impairment (Chao et al., 2010); therefore, rescue of LTP by mGlu_7_ potentiation is consistent with the proposed model by which mGlu_7_-mediated inhibition of GABA release is required for LTP at SC-CA1 synapses (Klar et al., 2015).

At the behavioral level, mGlu_7_ potentiation by intraperitoneal administration of the brain penetrant PAM, VU0422288, is able to improve performance in assays of cognition in RTT model mice (Gogliotti et al., 2017). While many studies in *Grm7^-/-^* mice have implicated a role for mGlu_7_ in learning and memory (Hölscher et al., 2005; Callaerts-Vegh et al., 2006; Goddyn et al., 2008), this is the first report of mGlu_7_ activity being modulated in a positive direction to reverse a deficit in cognition. VU0422288 is also able to increase performance in a social novelty task and reduce the number of apneas detected by whole body plethysmography (Gogliotti et al., 2017). These data suggest that mGlu_7_ potentiation may be a valid approach to address multiple RTT-associated symptom domains. It is important to note that these experiments used mice with a global deletion of *Mecp2*. As RTT is most commonly caused by *MECP2* point mutations in humans, it will be important to elucidate the effect of various point mutations on mGlu_7_ expression/function to identify patient subpopulations that would be predicted to benefit from an mGlu_7_ PAM.

mGlu_7_ has also been investigated for its therapeutic utility in a mouse of MDS. In contrast to RTT, MDS occurs when the region of the X chromosome containing *MECP2* is duplicated or triplicated, and is predicted to account for 1% of cases of unexplained X-linked intellectual disability (Lugtenberg et al., 2009). MDS patients present with infantile hypotonia, autism-associated symptoms, speech impairment, respiratory infections, and epilepsy (Ramocki et al., 2010). This disorder highlights the point that precise regulation of MeCP2 expression is required for normal brain function and that excess MeCP2 protein is detrimental. Fisher et al. (2017) tested whether mGlu_7_ protein levels are affected in *MeCP2-Tg1* mice, a model for MDS. Contrary to a hypothesis of bidirectional regulation, mGlu_7_ protein levels are unchanged in most brain regions in *MeCP2-Tg1* mice. Furthermore, neither genetic reduction of mGlu_7_ protein levels or administration of the mGlu_7_ NAM ADX71743 had any impact on anxiety and fear learning phenotypes in *MeCP2-Tg1* mice (Fisher et al., 2017). These findings suggest that that mGlu_7_ expression/function may only be affected by MeCP2 hypofunction and not overexpression. More studies are warranted to understand the molecular interaction between MeCP2 and mGlu_7_ expression. This information will inform future drug development of mGlu_7_ PAMs for RTT and other NDDs in which *MECP2* mutations have been identified.

## Conclusion

Neurodevelopmental disorders are a prevalent group of disorders with limited treatment options and mGlu_7_ represents one potential access point for pharmacological intervention. *GRM7* gene disruptions identified in patients with NDDs provide clinical rationale for this approach. Pre-clinical studies in rodent models suggest that decreased mGlu_7_ function is sufficient to mimic phenotypes that correlate to NDD symptom domains and that positive modulation of mGlu_7_ activity can improve some deficits, specifically in a mouse model of RTT. However, NDDs are highly heterogeneous and are likely the result of unique molecular pathologies that converge to produce similar circuit and behavioral phenotypes. Therefore, further studies are needed to identify and understand which subpopulations may benefit from an mGlu_7_-mediated therapy. In parallel, further development of improved tool compounds will facilitate studies focused on understanding mGlu_7_ receptor function in brain circuits and behaviors associated with NDDs.

## Author Contributions

NF and MS equally contributed to the first draft of this review. All authors read and edited the manuscript prior to submission.

## Conflict of Interest Statement

The authors declare that the research was conducted in the absence of any commercial or financial relationships that could be construed as a potential conflict of interest. The reviewer FG and handling Editor declared their shared affiliation.

## Abbreviations

ADHD: attention deficit hyperactivity disorder
ADX71743: (+)-6-(2,4-dimethylphenyl)-2-ethyl-6,7-dihydrobenzo[*d*]oxazol-4(5*H*)-one
ADX88178: 5-methyl-*N*-(4-methylpyrimidin-2-yl)-4-(1*H*-pyrazol-4-yl)thiazol-2-amine
AMN082: *N,N′*-dibenzhydrylethane-1,2-diamine dihydrochloride
ASDs: autism spectrum disorders
CaM: calmodulin
cAMP: cyclic adenosine monophosphate
CNS: central nervous system
EC50: effective concentration 50
ELFN1: extracellular-leucine-rich repeat fibronectin type III domain containing 1
EPM: elevated plus maze
GABA: γ-aminobutyric acid
GIRK: G protein inwardly rectifying potassium channel
GPCR: G protein-coupled receptor
GTPγS: guanosine 5′-O-[γ-thio]triphosphate
IC50: inhibitory concentration 50
ID: intellectual disability
L-AP4: L-2-amino-4-phosphonobutyric acid
LiCl: lithium chloride
LSP1-2111: (2*S*)-2-amino-4-[hydroxy[hydroxy(4-hydroxy-3-methoxy-5-nitro-phenyl)methyl]phosphoryl]butanoic acid
LSP2-9166: (2S)-2-amino-4-(((4-(carboxymethoxy)-3-(trifluoromethoxy)phenyl)(hydroxy)methyl)(hydroxy)phosphoryl)butanoic acid
LSP4-2022: (2*S*)-2-amino-4-({[4-(carboxymethoxy)phenyl](hydroxy)methyl}(hydroxy)phosphoryl)butanoic acid
LTD: long-term depression
LTP: long-term potentiation
LY341495: (2*S*)-2-amino-2-[(1*S*,2*S*)-2-carboxycycloprop-1-yl]-3-(xanth-9-yl)propanoic acid
MacMARCKS: macrophage myristoylated alanine-rich C-kinase substrate
MDS: *MECP2* Duplication syndrome
MeCP2: methyl-CpG binding protein 2
mGlu: metabotropic glutamate receptor
MMPIP: 6-(4-methoxyphenyl)-5-methyl-3-pyridin-4-ylisoxazolo[4,5-*c*]pyridin-4(5*H*)-one
MRG: mortality factor-related gene
NAM: negative allosteric modulator
NDDs: neurodevelopmental disorders
NK1: neurokinin-1 receptor
OLM: oriens-lacunosum-moleculare
PAM: positive allosteric modulator
PICK1: protein interacting with C kinase 1
PIH: phosphatidylinositol hydrolysis
PKA: protein kinase A
PKC: protein kinase C
PLC: phospholipase C
PTZ: pentylenetetrazole
RTT: Rett syndrome
SC-CA1: Schaffer Collateral-CA1
SLIN: stratum lucidum interneuron
SNP: single nucleotide polymorphism
VFD: Venus flytrap domain
VU6005649: 3-(2,3-difluoro-4-methoxy-phenyl)-2,5-dimethyl-7-(trifluoromethyl)pyrazolo[1,5-*a*]pyrimidine
VU6010608: 3,4-dimethoxy-*N*-[2-(1*H*-1,2,4-triazol-1-yl)-5-(trifluoromethoxy)phenyl]benzamide
XAP044: 7-hydroxy-3-(4-iodophenoxy)-4*H*-chromen-4-one
