# Corrigendum: Metabotropic Glutamate Receptor 7: A New Therapeutic Target in Neurodevelopmental Disorders

**DOI:** 10.3389/fnmol.2018.00444

**Published:** 2018-12-14

**Authors:** Nicole M. Fisher, Mabel Seto, Craig W. Lindsley, Colleen M. Niswender

**Affiliations:** ^1^Department of Pharmacology and Vanderbilt Center for Neuroscience Drug Discovery, Vanderbilt University , Nashville, TN, United States; ^2^Department of Chemistry, Vanderbilt University, Nashville, TN, United States; ^3^Vanderbilt Kennedy Center, Vanderbilt University Medical Center, Nashville, TN, United States

**Keywords:** Neurodevelopmental disorder, ASD, Rett syndrome, mGlu7, GRM7, allosteric modulator

In the original article, there was a mistake in Figure 1 as published. The chirality of L-AP4 and LSP1-2111 was incorrect. pEC_50_ values have also been corrected for LSP1-2111 in Table 1. The authors apologize for these errors and state that they do not change the scientific conclusions of the article in any way. The original article has been updated.

**Figure 1:**
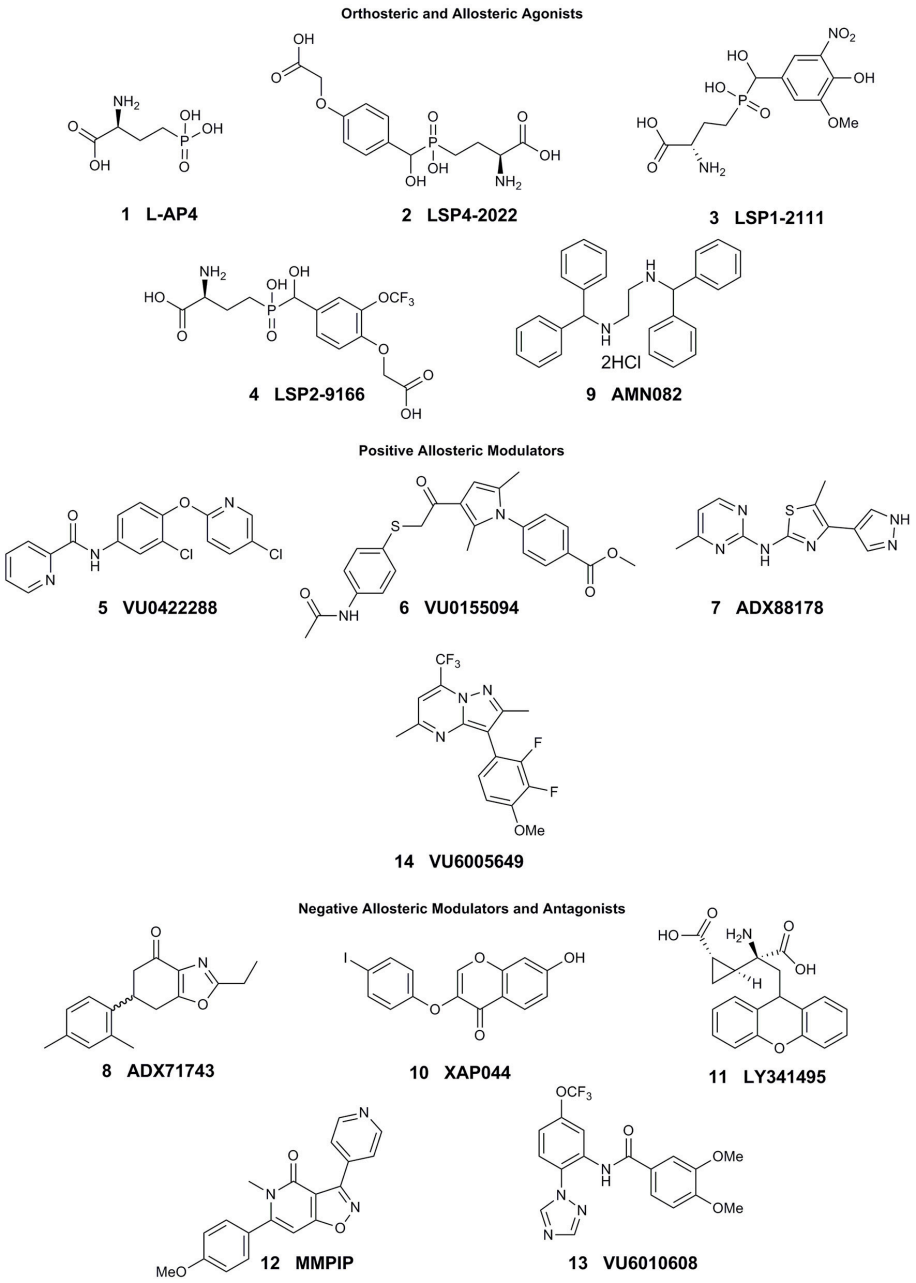
Current tool compounds used to study mGlu_7_.

**Table 1:** Summary of current tool compounds used to study mGlu_7_.

**Name (#)**	**Type**	**mGlu_**7**_ pEC_**50**_/pIC_**50**_**	**mGlu_**8**_ pEC_**50**_/pIC_**50**_**	**mGlu_**4**_ pEC_**50**_/pIC_**50**_**	**mGlu_**6**_ pEC_**50**_/pIC_**50**_**	**Source**
L-AP4 **(1)**	Orthosteric agonist	3.47 (PIH)	6.53 (PIH)	7.00 (PIH)	5.62 (PIH)	Acher et al., 2012; Selvam et al., 2018
		3.61 (Ca^2+^)	6.53 (Ca^2+^)	6.89 (Ca^2+^)	6.00 (Ca^2+^)
LSP4-2022 **(2)**	Orthosteric agonist	4.34 (Ca^2+^)	4.54 (Ca^2+^)	6.96 (Ca^2+^)	5.36 (Ca^2+^)	Acher et al., 2012; Goudet et al., 2012; Selvam et al., 2018
LSP1-2111 **(3)**	Orthosteric agonist	4.28 (PIH)	4.18 (PIH)	5.66 (PIH)	5.77 (PIH)	Selvam et al., 2018
		4.00 (Ca^2+^)	4.71 (Ca^2+^)	6.05 (Ca^2+^)	5.49 (Ca^2+^)
LSP2-9166 **(4)**	Orthosteric agonist	5.71 (Ca^2+^)	4.25 (Ca^2+^)	7.22 (Ca^2+^)	not reported	Acher et al., 2012
VU0422288 **(5)**	Group III PAM	6.85 (Ca^2+^)	6.93 (Ca^2+^)	6.98 (Ca^2+^)	not reported	Jalan-Sakrikar et al., 2014
VU0155094 **(6)**	Group III PAM	5.80 (Ca^2+^)	6.07 (Ca^2+^)	5.48 (Ca^2+^)	not reported	Jalan-Sakrikar et al., 2014
ADX88178 **(7)**	mGlu_4/8_ PAM	>4.52 (Ca^2+^)	5.66 (Ca^2+^)	8.46 (Ca^2+^)	>5	Le Poul et al., 2012
ADX71743 **(8)**	mGlu_7_ NAM	7.20 (human, Ca^2+^)	inactive	inactive	inactive	Kalinichev et al., 2014
		7.06 (rat, Ca^2+^)	inactive	inactive	inactive
AMN082 **(9)**	Allosteric agonist	6.59 (GTPγS)	>5 (GTPγS)	>5 (GTPγS)	>5 (GTPγS)	Mitsukawa et al., 2005
XAP044 **(10)**	Antagonist	5.26 (cAMP)	4.48 (cAMP)	inactive	inactive	Gee et al., 2014
		5.55 to 5.46 (GTPγS)			
LY341495 **(11)**	Orthosteric antagonist	6.00 (cAMP)	6.76 (cAMP)	4.66 (cAMP)	not reported	Kingston et al., 1998
MMPIP **(12)**	mGlu_7_ NAM	6.66 (cAMP)	>5 (cAMP)	>5 (cAMP)	not reported	Suzuki et al., 2007
		7.15 (Ca^2+^)				Niswender et al., 2010
		6.14 (Thallium)				Niswender et al., 2010
VU6010608 **(13)**	mGlu_7_ NAM	6.12 (Ca^2+^)	>5 (Ca^2+^)	>5 (Ca^2+^)	inactive (>5)	Reed et al., 2017
VU6005649 **(14)**	mGlu_7/8_ PAM	6.19 (Ca^2+^)	5.59 (Ca^2+^)	>5 (Ca^2+^)	inactive	Abe et al., 2017

*NAM, negative allosteric modulator; PAM, positive allosteric modulator; EC_50_, effective concentration 50; IC_50_, inhibitory concentration 50. Assay type is indicated in parentheses: PIH, Phosphatidylinositol hydrolysis; cAMP, cAMP accumulation; Ca^2+^, Calcium mobilization; GTPγS, GTPγS binding*.

## Conflict of Interest Statement

The authors declare that the research was conducted in the absence of any commercial or financial relationships that could be construed as a potential conflict of interest.
